# CrossLink: a novel method for cross-condition classification of cancer subtypes

**DOI:** 10.1186/s12864-016-2903-z

**Published:** 2016-08-22

**Authors:** Chifeng Ma, Konduru S. Sastry, Mario Flore, Salah Gehani, Issam Al-Bozom, Yusheng Feng, Erchin Serpedin, Lotfi Chouchane, Yidong Chen, Yufei Huang

**Affiliations:** 1 Department of Electrical and Computer Engineering, University of Texas at San Antonio, San Antonio, TX USA; 2 Weill Cornell Medicine-Qatar, Doha, Qatar; 3 Hamad Medical Corporation, Doha, Qatar; 4 Department of Mechanical Engineering, University of Texas at San Antonio, San Antonio, TX USA; 5 Department of Electrical and Computer Engineering, Texas A&M University, College Station, TX USA; 6 Department of Epidemiology and Biostatistics, University of Texas Health Science Center at San Antonio, San Antonio, TX USA; 7 Greehey Children Cancer Research Institute, University of Texas Health Science Center at San Antonio, San Antonio, TX USA; 8 Division of Translational Medicine, Sidra Medical and Research Center, Doha, Qatar

## Abstract

**Background:**

We considered the prediction of cancer classes (e.g. subtypes) using patient gene expression profiles that contain both systematic and condition-specific biases when compared with the training reference dataset. The conventional normalization-based approaches cannot guarantee that the gene signatures in the reference and prediction datasets always have the same distribution for all different conditions as the class-specific gene signatures change with the condition. Therefore, the trained classifier would work well under one condition but not under another.

**Methods:**

To address the problem of current normalization approaches, we propose a novel algorithm called CrossLink (CL). CL recognizes that there is no universal, condition-independent normalization mapping of signatures. In contrast, it exploits the fact that the signature is unique to its associated class under any condition and thus employs an unsupervised clustering algorithm to discover this unique signature.

**Results:**

We assessed the performance of CL for cross-condition predictions of PAM50 subtypes of breast cancer by using a simulated dataset modeled after TCGA BRCA tumor samples with a cross-validation scheme, and datasets with known and unknown PAM50 classification. CL achieved prediction accuracy >73 %, highest among other methods we evaluated. We also applied the algorithm to a set of breast cancer tumors derived from Arabic population to assign a PAM50 classification to each tumor based on their gene expression profiles.

**Conclusions:**

A novel algorithm CrossLink for cross-condition prediction of cancer classes was proposed. In all test datasets, CL showed robust and consistent improvement in prediction performance over other state-of-the-art normalization and classification algorithms.

## Background

The rapid development of high-throughput technologies including microarray and high-throughput sequencing have significantly advanced our understanding of disease including cancer [1]. Torrent of gene expression profiles from cancer cell lines and patient samples have been and are being made available by efforts ranging from large group projects such as The Cancer Genome Atlas to individual labs [2–4]. Significant efforts have been devoted to developing new genomic approaches using gene expression and other genomic data for cancer diagnosis and prognosis [5]. As exciting new results generated from these research efforts continue to challenge our knowledge of cancer, these efforts are also poised to revolutionize the practice of cancer therapy. A large number of gene expression based biomarkers such as PAM50 have been reported to improve cancer classification and prediction of therapy response [6–10].

As exciting as these new discoveries are, their translation from laboratories to real clinical practice remains a challenge. Overcoming systematic and condition-specific biases presented in expression data as a result of different technological platforms, varying experimental/measurement conditions, and heterogeneities in the patient age, gender and race continues to be an issue yet to be completely addressed. Although improved standards in uniform experimental and clinical protocols have and will help reduce the systematic biases, eliminating biases specific to experimental/clinical conditions, patient individuals, technology/platforms would be more effective dealt with by using computational algorithms. The well-known Microarray Quality Control project (MAQC) spearheaded the algorithm development in this front and demonstrated that through careful algorithm-based normalization, consistently differentially expressed genes can be reproduced in data produced from different platforms [11]. Since then, many algorithms have been reported to address different aspects of cross-platform data normalization [12–17]. However, removing biases from different platforms might require using different normalization algorithms. Furthermore, the problem of mitigating condition-specific bias due to differences in experimental/clinical conditions and patient characteristics has not been given sufficient attention. Therefore, a normalization algorithm may work well under one condition but not under another [12].

**Fig. 1:**
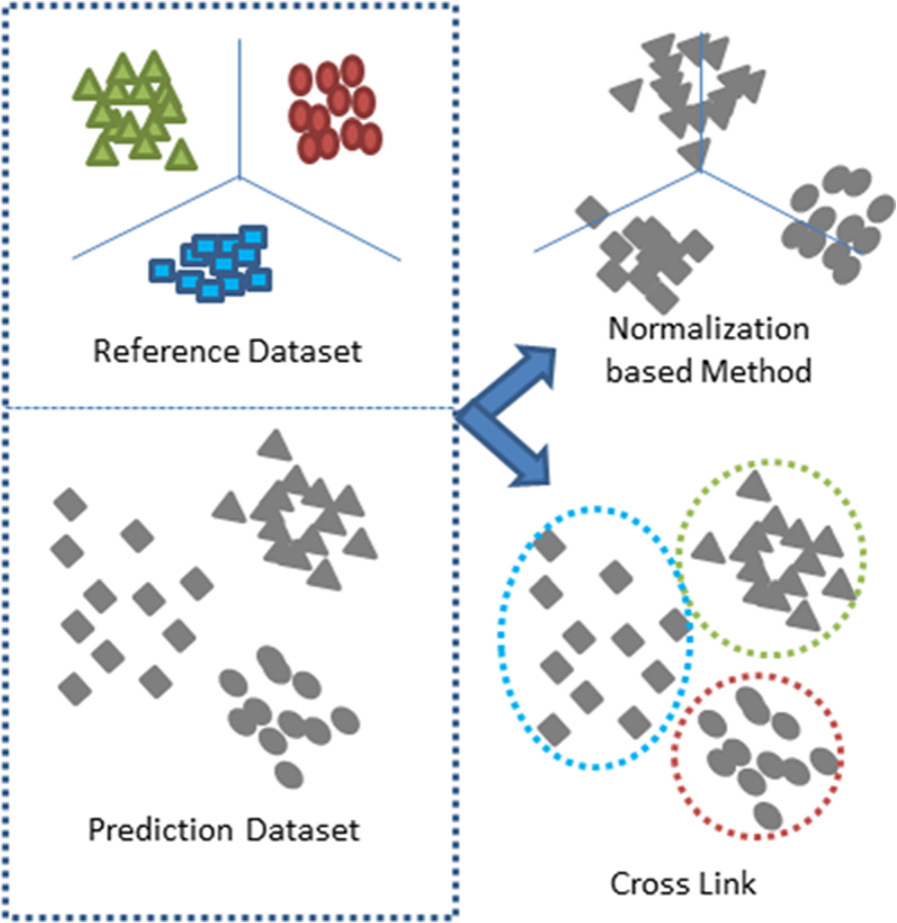
General idea of CL. Due to condition-specific biases, the existing normalization algorithm might fail to normalize the distributions of class-specific gene signatures in the reference and prediction datasets. Therefore, the classifier trained using the reference dataset would not work well for the prediction dataset (top right figure). Unlike normalization based approach, CL exploits the fact that the signature is unique to its associated class under any condition and thus employs an unsupervised clustering algorithm to discover this unique signature, hence the class label (bottom right figure)

In this paper, we consider the problem of predicting cancer classes (e.g. subtypes) based on patient gene expression profiles. Particularly, a reference expression dataset is assumed available, where the true cancer class labels for each sample are known. However, compared with the reference dataset, the prediction dataset is generated using a different platform, from patient samples of, for instance, different races, and collected under a different condition. That is, we assume that the prediction dataset contains both systematic and condition-specific biases. Currently, the mainstream practice to this prediction starts by first normalizing the reference and prediction dataset so that both can follow the same desired characteristics (e.g. distribution). Then, a classifier is trained using the normalized reference dataset, which would produce a set of signature genes, accompanied also by their associated class-specific expression signatures [14]. This gene-signature based classifier is finally applied for cancer class prediction in the prediction dataset. The premise for the trained classifier to work well is that the distributions of the label-specific gene signatures in the reference and prediction datasets should remain similar after normalization. However, when both systematic and condition-specific biases are present in data, it cannot be guaranteed that a normalization algorithm can map the gene signatures in the reference and prediction datasets to have the same distribution for all different conditions. As a result, the trained classifier would fail under a different condition (Fig. 1), where one will have to train a new classifier after applying a different normalization algorithm.

To address the problems of current normalization based approaches, we propose a novel algorithm called CrossLink (CL). The CL algorithm represents a complete departure from the current normalization-classification paradigm. CL only assumes that each cancer class is associated with a set of signature genes, which are independent of the conditions. However, CL recognizes that although for a specific condition, the signature genes should define a unique, cancer class-specific gene expression signature but this signature changes under a new condition. Moreover, the change in the signature is condition-specific and there is no universal, condition-independent normalization mapping of signatures. As a result, unlike existing normalization-based algorithms, CL does not attempt to explore a mapping of the signatures across different conditions; in contrast, it exploits the fact that the signature is unique to its associated class under any condition and thus employs an unsupervised clustering algorithm to discover these unique signatures (Fig. 1).

The rest of the paper is organized as follows: In Methods, the workflow of CL is discussed in details. In Results, we demonstrate the improved, robust performance of CL using both simulated and real data. The concluding remarks are drawn in Conclusion.

## Methods

### Problem definition and CL algorithm details

Suppose that we are given a reference dataset that measures global gene expression of a set of known cancer classes (e.g., PAM50 subtypes). The problem that CL addresses is to predict the cancer classes for a set of new expression data samples collected under a different condition. The workflow of the CL algorithm can be divided into two steps: signature gene set identification and class prediction. For the first step, the goal is to identify the signature gene sets for each cancer classes from the reference dataset. For the second step, the signature gene sets are used to predict the class labels for the prediction dataset. The details of these two steps will be discussed next. Notice that before implementing CL, gene entries of data samples from reference and prediction datasets need to be mapped into a set of common reference entries. By default, CL uses the common Gene Symbol as the reference entries. A data entry is removed from all samples if no entry in any samples can be mapped.

#### Signature gene set identification

As commonly defined, the signature gene set of a cancer class include genes that show uniquely differential expression in that class. Analysis of Variance (ANOVA) solves such problem. Suppose that there are *N* classes and the reference dataset contains *M* genes. A one-way ANOVA model is then proposed for each gene expression

1 $$ {G}_i={g}_i+{\alpha}_k+\epsilon $$

where *k* = 1 … *N*, *G*_*i*_ is the gene expressions of the ith gene in all samples, *g*_*i*_ is the ambient expression of this gene while α_*k*_ represents the unique effect of the *k*th class on the gene expression, and *ε* ~ N(0, σ) denotes the zero-mean noise. The ANOVA analysis identifies these signature genes for each class by testing the hypothesis

2 $$ {\mathrm{H}}_0:\mathrm{all}\kern0.3em {\alpha}_k=0 $$

against the alternative hypothesis

3 $$ {\mathrm{H}}_1:\mathrm{at}\kern0.3em \mathrm{least}\kern0.3em \mathrm{one}\kern0.3em {\alpha}_k\ne 0. $$

A multiple comparison procedure such as Benjamini-Hochberg is applied to calculate the False Discovery Rates (FDRs) for each gene and the genes that are tested significant (FDR>0.05) for a class are determined as the candidate gene of the signature gene set of that class. An additional filtering step is followed to reduce the possible false positive signature genes. The filtering imposes three expression conditions on every candidate signature genes, first, the candidate signature gene should have the largest absolute average expression in the class it test significant for; second, a lower limit is introduced on the difference of average expression between the class it tested significantly for and the rest; and third, a lower limit is introduced on the absolute average expression of the class it is testing. The leave-one-out cross-validation was applied to determine the limits that yield the best classification outcomes. Only those candidate genes that satisfy all three conditions will be retained to form the final signature gene sets. In the end, *N* signature gene sets will be obtained.

#### Class prediction

Once the signature gene sets are determined for each class, the next step is to predict the class labels for a new set of data samples. As opposed to using a supervised approach that performs the prediction with a classifier trained on the (normalized) training data set, CL employs a novel unsupervised solution. Since we assume that each signature gene set possesses a unique expression signature for the corresponding class, it would be natural to expect that the class-specific gene set can separate the dataset into two groups: one that belongs to the target class that the gene set is associated with and the other one that contains samples from other classes. Therefore, CL employs the K-means clustering [18] to group the dataset into two clusters and this is performed for each of the *N* signature gene sets independently. For each of the clustering results, the cluster with a higher absolute average expression value is determined target class, whereas the other cluster is determined as the non-target class. Now that each sample can be assigned with a target class label for any of the *N* classes, a sample can be associated with multiple class labels. To resolve this ambiguity, a multiple call adjustment procedure is proposed. Specifically, for each class *j* that is assigned to a sample *i*, a confidence score *S*_*i,j*_ is calculated as:

4 $$ {S}_{i,j}={p}_1\ast {p}_2 $$

Where

5 $$ {p}_1=- \log \left(\mathrm{P}\left({G}_j\Big|{\mu}_{j, nt},{\sigma}_{j, nt}\right)\right) $$

6 $$ {p}_2=- \log \left(\mathrm{P}\left({\mu}_{j,t}-{\mu}_{j, nt}\Big|{\mathrm{t}}_{0,{s}_p,{n}_1+{n}_2-2}\right)\right) $$

where* j* = 1, …, *k*. Since for each class specific signature gene set, the clustering algorithm separates the dataset into two clusters: class target clustering and class non-target cluster. We further assume that the class target cluster can be modeled by a normal distribution N_1_(*μ*_*j,t*_, *σ*_*j,t*_) and the non-target cluster by another normal distribution N_2_(*μ*_*j,nt*_, *σ*_*j,nt*_). The first criteria *p*_1_ calculate the probability of samples in the class *j*’s gene set under the hypothesis that the non-target cluster distribution is true. This probability essentially measures the distance from the sample to the center of the class non-target cluster center. The second criteria *p*_2_ calculate the probability of *μ*_*j,t*_ − *μ*_*j,nt*_ under the hypothesis that the difference of two means follows a student *t* distribution $\mathrm{t}\left({t}_{0,{s}_p,{n}_1+{n}_2-2}\right)$ where means is 0, variance *s*_*p*_ is the pooled variance of two normal distribution N_1_ and N_2_, and degree of freedom is *n*_1_ + *n*_2_ − 2, where *n*_1_ and *n*_2_ are the sample size of class target cluster and class non-target cluster respectfully. This probability is essentially a two-sample *t* test, measures the distance between the class target cluster and class non-target cluster. It is obvious that both two criteria are maximized to yield a higher confidence level. A confidence score is determined by multiplying these two criteria together. The class with the highest confidence will be assigned to the sample

7 $$ {\mathrm{Label}}_i={\mathrm{argmax}}_j\kern0.2em \left({\mathrm{S}}_{i,j}\right) $$

### A metric for evaluating PAM50 subtype prediction using ER and PR status

We investigated CL performance for cross experiment predictions of PAM50 subtypes (See Results for details). However, the true PAM50 subtypes are rarely available for most of the prediction datasets and when this is the case, direct evaluation of the CL performance is infeasible. In contrast, the pathological biomarker assessments of the estrogen receptor (ER) progesterone receptor (PR) are much more accessible for most of the patient samples. Particularly, in a recent study, over 800 breast cancer patients were genetically profiled and their PAM50 subtypes tested by a novel RT-qPCR approach that is independent of microarray platform and their ER and PR status were recorded [19]. This study inspired us to propose an indirect evaluation of the PAM50 classification result by seeking a link between the ER-PR markers status and PAM50 subtypes. Table 1 tallies the distribution of patients from this study over a classification based on both ER-PR status and PAM50 subtypes. Based on Table 1, the empirical conditional probability of each PAM50 classification given an ER-PR status, or P(PAM50|ER, PR) can be calculated, which can be used as the confidence level of predicting a PAM50 subtype given its ER-PR status. For example, if a patient was ER+ and PR+, then from Table 1, we can infer that our confidence of PAM50 prediction as the subtype LumA is 45.64 %. Notice that another important assessment HER2-status is also available and could be included into our analysis, but it is not as commonly documented as ER and PR. Because of this reason Her2 status is not included in our assessment. However, including Her2 could further improve the performance and is very straight forward as we explained. Over all, in the absence of true PAM50 labels, we propose the Indirect Summed Evaluation Probability (ISEP) to evaluate the PAM50 prediction results and ISEP is calculated as

**Table 1:** Distribution of patients on PAM50 subtypes and ER-PR status

	LumA	LumB	Her2	Basal	Normal
ER+,PR+	246	188	78	4	23
ER+, PR-	12	51	33	3	6
ER-, PR+	15	5	3	4	1
ER-, PR-	4	17	60	59	2

8 $$ \mathrm{ISEP}={\displaystyle {\sum}_{n=1}^N\left(\mathrm{PAM}{50}_n\Big|{\mathrm{ER}}_n,{\mathrm{PR}}_n\right)} $$

where *N* represents the size of the prediction dataset. Since different dataset certainly have different PAM50 class label rates, this difference in the class label rates could yield an accidentally equal ISEP. Also, because the conditional probability of each PAM50 class does not equal to each other, although the ISEPs of two experiments may differ, they could infer the exact same classification accuracy. Because of these reasons, we want to point out that the ISEPs for two datasets should not be compared.

### Code implementation and development environment

All algorithms are designed and implemented under Matlab R2013a. Function ‘anova1’ is used in the signature selection; function ‘kmeans’ is used in the classification procedure. The designed algorithm is also implemented with R (version 3.1.1). The R code and an example demonstrating the whole pipeline are provided to show how to extract signatures from a reference dataset and how they can be used to classify independent cross-condition samples. The package can be downloaded from http://compgenomics.utsa.edu/CrossLink/CL_R.zip.

### Data collection for Arabic breast cancer patients

The study was approved by the Institutional Review Board of Weill Cornell Medicine-Qatar and the Hamad Medical Corporation’s Ethics Committee, Doha. All subjects signed informed consent documents for participation in this study. The diagnosis of cancer was confirmed by histopathologic analyses. Expression of ER, PR and Her2 was revealed by immunohistochemistry. Human breast cancer tumor samples and non-malignant healthy breast tissues were collected, immediately placed in RNAlater solution and frozen at -80 °C until further use. RNeasy Minikit (Qiagen) was used to extract and purify RNA from these breast tissue samples. The GeneChip Human Genome U133A 2.0 Array (Affymetrix) was used to explore the differentially expressed genes according to manufacturer’s instructions.

## Results

This section is separated into three parts: (1) the ability of CL for PAM50 classification is first demonstrated in several scenarios; (2) the application of CL on Cancer2000 classification is then demonstrated; (3) a Qatar breast cancer patients’ Microarray data analysis is conducted.

### Cross-experiment prediction of PAM50 breast cancer intrinsic subtype

PAM50 breast cancer intrinsic subtype is a gene expression based classification system that includes five breast cancer subtypes: Luminal A (LumA), Luminal B (LumB), Her2 enriched (Her2), Basal and Normal-breast like (Normal) [20]. It has been well studied and has the ability to predict patient’s survival [19, 21, 22]. The PAM50 system is also accompanied by a 50-gene based classifier for subtype prediction based on an expression data. However, the usage of this classifier requires the prediction datasets to be also generated from the same platform as that of PAM50 (Agilent Human 1A Oligo Microarrays). Otherwise the prediction accuracy would suffer significantly due to platform bias [23]. This limitation underscores the need for a system that can faithfully map the PAM50 classification to samples generated from a different platform.

#### PAM50 prediction of a simulated dataset

We first evaluated CL on a simulated dataset, where the true class labels for the test samples are known. Breast Cancer Patient Microarray dataset (BRCA) from The Cancer Genome Atlas (TCGA) [24] was used in this experiment. This dataset includes over 500 microarray samples as well as detailed clinical information of breast cancer patients. BRCA dataset also includes PAM50 subtypes for each sample. This dataset will be used as the reference dataset for all PAM50 prediction cases. To simulate a cross-experiment prediction, a five-fold cross-validation scheme was implemented, where in each cross-validation, the four folds of dataset was considered as the reference set and the other one fold was used as the prediction set. To simulate the effect of the cross-experiment bias in the prediction set, the experimental bias was added to each gene expression value G_ij_ according to the following model:

9 $$ {G}_{ij}={g}_{ij}+{\alpha}_i+{\epsilon}_{ij} $$

where *G*_*ij*_ is the gene expression of the *i*th gene in the *j*th sample of the prediction dataset, *α*_*i*_ ~ N(0, *σ*^2^) is the experimental bias for gene *i* and is constant across all the samples, and *ε*_*ij*_ ~ N(0, *σ*_1_^2^) models the sample-specific noise. Notice that the experimental biases are different for different genes. These gene-specific biases simulates the varying influence of a different experimental condition on the expression of different genes. In this experiment, we investigated the robustness of CL prediction against experimental bias, where we let *σ*^2^ equal to 0.5 and *σ*_1_^2^ ranged from 0 to 7.

**Fig. 2:**
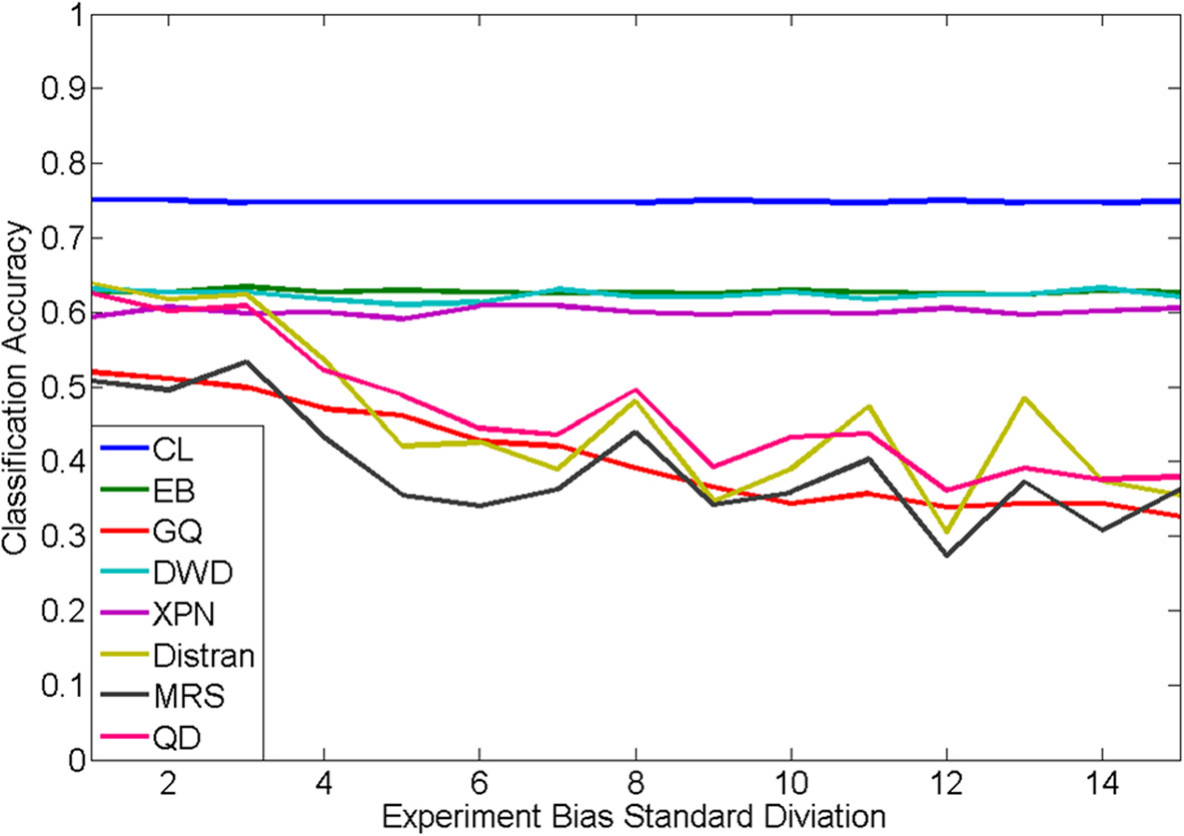
Comparison of CL and seven cross platform normalization + SVM algorithms for PAM50 classification accuracy. Horizontal line represents the level of experimental bias level and vertical line represents the classification accuracy

The prediction performance of CL and seven state-of-the-art cross- platform normalization algorithms are shown in Fig. 2. These seven algorithms include Cross-Platform Normalization (XPN) [12], Distance Weighted Discrimination (DWD) [13], Empirical Bayes (EB) [14, 15], Median Rank Scores (MRS) [14], Quantile Discretization(QD) (Warnat, et al., 2005), Distribution Transformation(DisTran) [16], and Gene Quantiles (GQ) [17]. For each algorithm, a Support Vector Machine (SVM) based one-vs-the-rest multi-class classification algorithm was applied to the normalized data for class label prediction. In order to keep the genes used in our CL to be the same as those in SVM to obtain a fair comparison, SVM was applied on the pooled gene signature set obtained in the CL procedure. Overall, CL produced the best prediction performance at all bias levels. Interestingly, even at no bias, CL outperformed all seven other normalization algorithms, where CL obtained a classification accuracy of 0.75, which improved 0.13 percentage points over the best performing normalization algorithm (DisTran at 0.6393). The reason of this could because that the normalization algorithms actually introduced more artificial bias into the system because it assumes there was bias between training and testing datasets. Moreover, the performance of CL remained robust against the increase of the experiment biases. In contrast, four of the seven normalization algorithms suffered different degree of performance degradation with the increase of the experimental bias. Taken together, these results suggest that CL not only can obtain improved performance when no experimental bias present, but is also immune from the influence of constant, gene-specific experimental bias.

#### PAM50 prediction for the UNC breast cancer dataset

We carried out next an evaluation of CL performance on a real dataset: the UNC breast cancer patient dataset. In this case, the PAM50 subtypes are available and the prediction performance can be directly evaluated. The data samples were collected from Gene Expression Omnibus (GEO) under the data entry GSE2740 [25]. Out of all samples from the entire dataset, 349 samples from the platform GPL1390 were extracted. We used the TCGA-BRCA dataset as the reference dataset. The signature gene sets for each PAM50 subtypes (Table 2) were obtained in the signature gene sets identification step of CL.

**Table 2:** The size of CL selected gene set for PAM50 classification

Subtype	Selection gene size
LumA	60
LumB	60
Her2	63
Basal	299
Normal	52

In this process, the impact of different threshold (see Methods for details) was also investigated (Table 3). We can see that there is no significant trend in T1 and T2 vs. the classification performance. Because of this, the best way to locate a combination that yields the best classification performance would still be through a gradient search for a given range. In this case, two threshold were both given a range of (0.1, 1) and the combination (0.1, 0.8) was chosen for the best leave one out classification accuracy and the corresponding gene signature was obtained.

**Table 3:** Impact of different threshold on selected size, value and corresponding classification accuracy

T1 T2 combination	Selected gene size	Smallest absolute expression	Classification accuracy
0.1, 0.1	790	0.21	79.66 %
0.3, 0.1	637	0.28	74.02 %
0.5, 0.1	441	0.37	72.99 %
0.7, 0.1	292	0.48	72.99 %
0.9, 0.1	189	0.66	74.53 %
1.1, 0.1	123	0.73	63.42 %
0.3, 0.3	634	0.30	74.02 %
0.3, 0.5	600	0.50	75.56 %
0.3, 0.7	532	0.70	73.85 %
0.3, 0.9	442	0.80	70.09 %
0.1, 0.8 (selected)	534	0.80	80.00 %

**Fig. 3:**
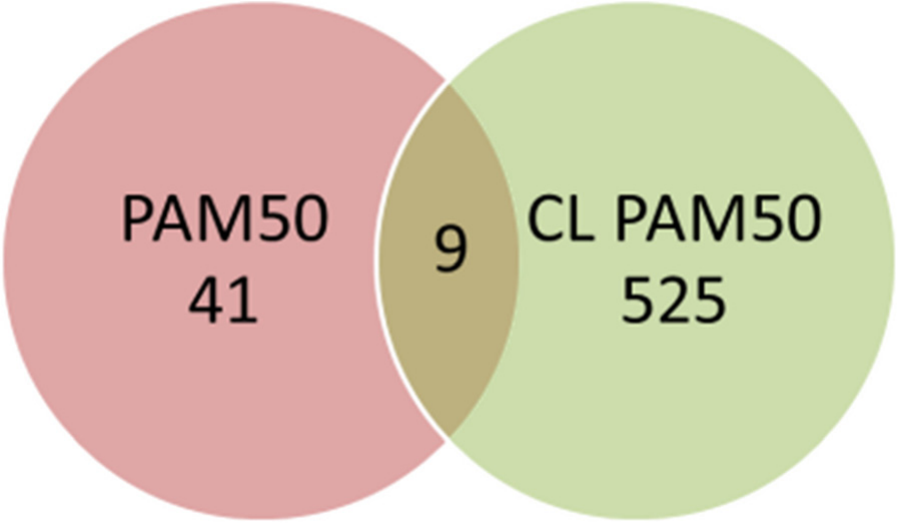
Comparison of CL selected PAM50 signature and PAM50 signature

This signature gene set yielded a leave one out classification rate of 80 % for the BRCA dataset. In addition, this gene set was pooled together and compared with the well-known PAM50 signature gene set (Fig. 3). Specifically, 9 genes are shared between PAM50 and CL, while the rest of the two gene set are completely different. This result suggests that while PAM50 signature gene set shows well established ability for subtype prediction in the expression pattern based algorithms [26], for some specific subtypes, the discriminative power of these genes are not as strong as CL selected gene set. The gene sets were then used in the subtype prediction step. Notice that TCGA-BRCA was also generated from the platform GPL1390, so there is no cross-platform biases. The prediction results are shown in Table 4, where CL achieves 73 % classification accuracy, which is a 16-percentage-point improvement over the best normalization algorithm (XPN: 55 %).

**Table 4:** Classification accuracy of PAM50 classification of GSE2740

Algorithm	Accuracy
CL	73 %
EB	55 %
GQ	55 %
DWD	56 %
XPN	57 %
DisTran	53 %
MRS	57 %
QD	56 %

#### PAM50 prediction for a dataset with no true PAM50 labels

**Fig. 4:**
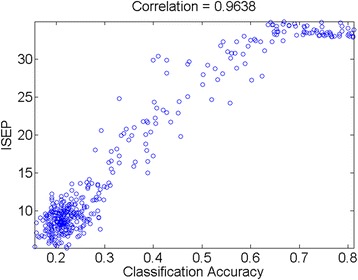
Classification accuracy vs ISEP for simulation case. Horizontal axis represents the classification accuracy and vertical axis represents the corresponding ISEP

**Fig. 5:**
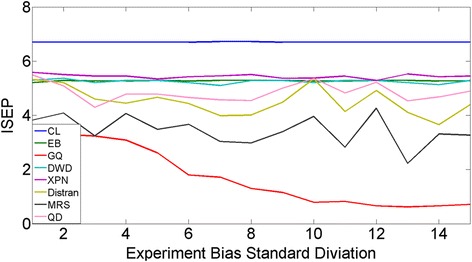
Plot of ISEP with experimental bias for CL and seven cross platform normalization algorithm + SVM in the Simulation Case. Horizontal axis represents the experiment Bias level and vertical axis represents the ISEP values

We then proceeded to test CL on additional datasets. This time, the true PAM50 labels were not available and we applied the proposed ISEP instead to direct assess the prediction performance. Before we proceeded to prediction, we first evaluated the relationship between the ISEP accuracy and the accuracy based on true PAM50 labels. The better the ISEP represents the true performance, the more correlated the ISEP and the true accuracy should be. ISEPs corresponding to different PAM50 classification accuracy based on the reference dataset (TCGA-BRCA) were calculated. The result shows that ISEP strongly correlated with PAM50 classification accuracy with a correlation coefficient of 0.96 (Fig. 4). The ISEPs in the previous simulation case were also calculated (Fig. 5). The result shows almost the same trend as the accuracy plot in Fig. 2. The average correlation coefficient between classification accuracy and its corresponding ISEP is 0.83. Overall, the result indicates that without the true PAM50 labels, ISEP could be used to evaluate the performance of PAM50 classification.

Next, CL was applied to a dataset that includes 28 breast cancer patients, whose stroma and epithelium cells were profiled with Affymetrix U133A 2.0 GeneChips (GSE10797). Only 20 samples with both ER and PR information were selected in order to calculate the ISEP accuracy. TCGA-BRCA was still used as the reference dataset and this time there was also a difference in platforms in addition to the apparent experimental differences. As a comparison, the original PAM50 classifier (R code) [27] was also applied in addition to the seven normalization algorithms. ISEP accuracies of each prediction outcomes were calculated and the results are summarized in Table 5. CL greatly outperforms all algorithms except QD, which has a slightly higher ISEP than CL (QD: 5.71 vs CL: 5.67). Particularly, the original PAM50 classifier greatly suffered from the platform bias and only achieved an ISEP of 3.3, which is the worst performance among all. Taken together, the results from this test and that on UNC breast cancer dataset confirm the improved performance of CL for cross-experiment predictions.

**Table 5:** ISEP of PAM50 prediction for CL and seven cross platform normalization algorithms + SVM for GSE10797

Algorithm	ISEP
CL	5.67
EB	3.61
GQ	3.86
DWD	3.66
XPN	4.09
DisTran	5.27
MRS	5.12
QD	5.71
PAM50	3.3

### Cross-experiment prediction of cancer 2000 subtypes

Recently, over 2000 breast cancer patients (cancer2000) were profiled and a classification including 10 novel breast cancer subtypes were reported based on the integrative study of microarray gene expression, copy number variation as well as gene mutation information [28]. These novel subtypes were shown to be associated with distinct patient survival. Since Cancer2000 subtypes were recently introduced, the perdition of Cancer2000 subtypes for other patient data has not yet been extensive studied. Given this interest, we investigated how CL performed in predicting Cancer2000 subtypes.

#### Evaluation by simulation

**Fig. 6:**
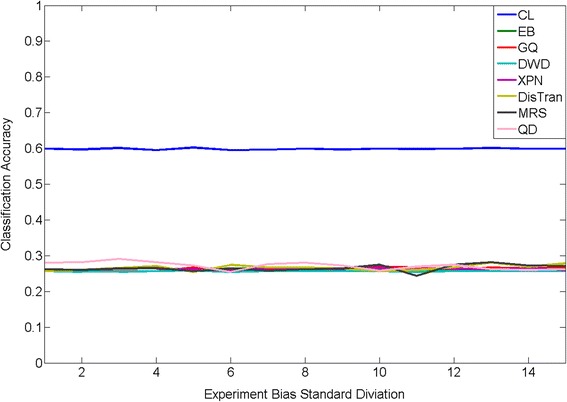
Comparison of Cancer 2000 Classification between CL and seven cross platform normalization algorithm + SVM in the simulation case. Horizontal axis represents the experimental bias level and vertical axis represents the classification accuracy

Cancer 2000 contains two parts, where first part is a discovery dataset that includes 997 breast cancer patients samples and the second part includes 5 additional validation sets including another over 900 breast cancer patient samples. For each patient, the expression levels of 48,803 genes were measured [28]. Here we used the discovery dataset as our reference dataset for all cancer 2000 subtype classification. The same procedure as in PAM50 was conducted and 10 signature gene sets were selected by CL for all 10 classes (Table 6). As for cancer2000 prediction, we first evaluated the CL performance on the cancer2000 dataset through 5-fold cross-validation and simulation, where the same model as in (1) was applied to model the experimental bias. Notice that the prediction problem is a 10-class classification and it is extremely challenging even without any experiment bias. Once again, CL significantly outperformed all normalization algorithms at all bias levels, registering a more than 100 % improvement in prediction accuracy (~0.6 for CL vs <0.3 for others; Fig. 6). The fact that none of the normalization algorithms achieved classification accuracy higher than 30 % at any bias levels speaks for the difficulty of this classification problem and also underscores the significance of the improvement that CL achieved.

**Table 6:** CL selected signature gene set size for cancer 2000

Subtype	Selection gene size
Class 1	367
Class 2	3111
Class 3	98
Class 4	207
Class 5	981
Class 6	501
Class 7	265
Class 8	247
Class 9	773
Class 10	286

#### Prediction of cancer2000 subtypes for TCGA-BRCA dataset

**Fig. 7:**
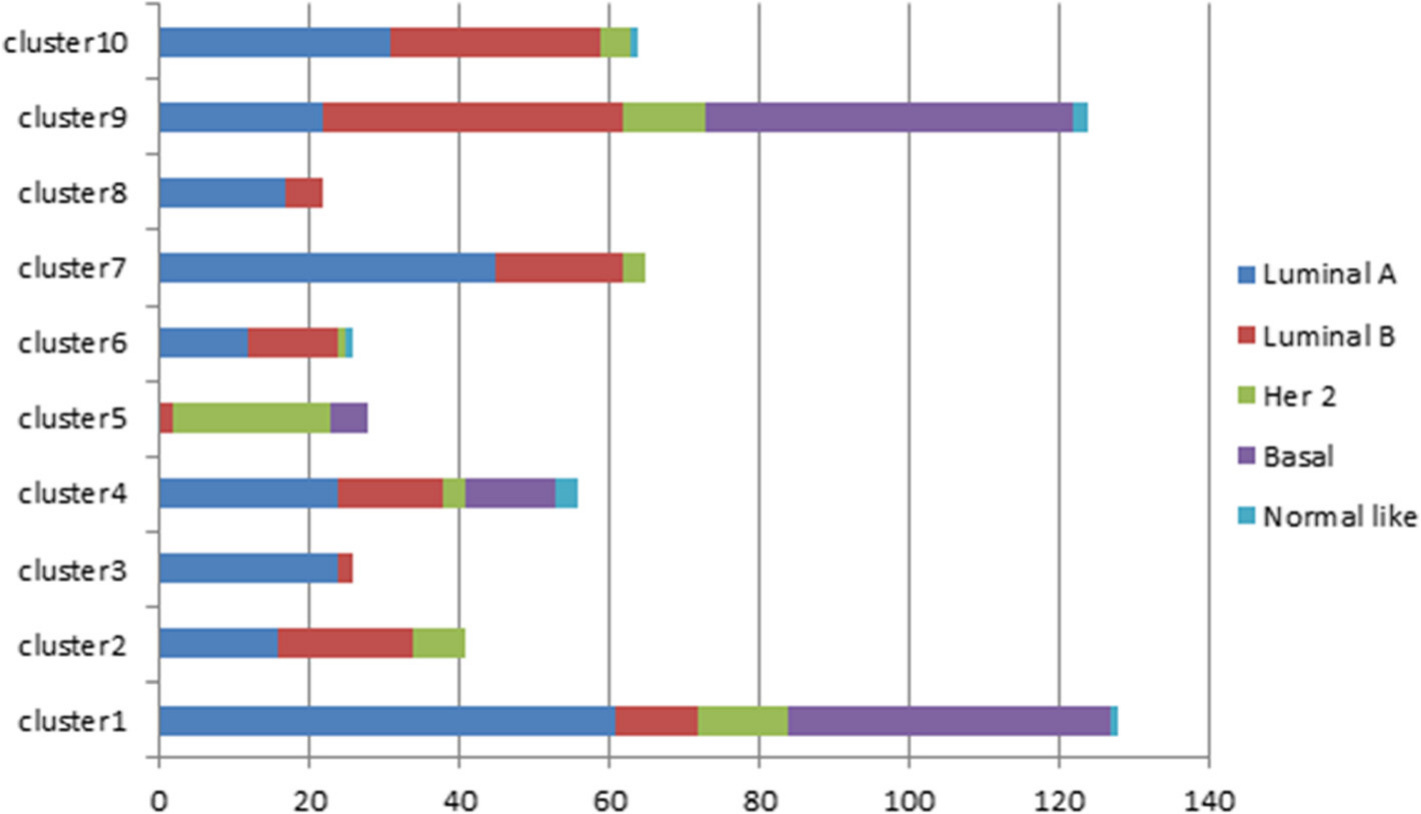
Cancer2000 classification for TCGA-BRCA dataset. Horizontal axis represents the number of samples classified for each cancer 2000 cluster. Different colors label the PAM50 class label

We then used CL to predict the Cancer2000 subtypes for TCGA-BRCA dataset. A set of 10 signature gene sets was first obtained on the reference Cancer2000 dataset (Table 6) and the prediction results were shown in Fig. 7. Although there was no true Cancer2000 classification for TCGA-BRCA samples, it was shown in [28] that the 10 subtypes have unique characteristics in terms of their protein marker status, PAM50 classification, mutation and copy number variation and these characteristics provide ample evidence to assess the performance. Here we selected 4 classes with characteristics available in BRCA dataset (Table 7). Using these characteristics, we evaluated the classification performance by assessing the enrichment of the characteristics in the corresponding class. The rest 6 classes were excluded because the corresponding characteristics were not available in the BRCA dataset. The analysis results of CL predictions and the seven normalization algorithms are presented in Table 8. It is clear that the Cancer2000 characteristics are highly enriched in CL predictions. For instance, 36 of 41 patients that were predicted as Class 2 by CL are ER positive. This is highly consistent with the fact that Class 2 is mainly characterized as ER positive (Table 7). Moreover, while Class 3 is mostly Luminal A samples, 24 of 26 Class 3 samples predicted by CL are Luminal A samples. Also, Class 5 includes mostly ER negative and HER2 enriched samples and among 28 CL identified Class 5 samples, 20 samples are ER negative and 21 samples are HER2 enriched. Similarly, Class 6 samples are enriched by ER positive and Luminal samples; 26 CL identified samples are all ER positive and 24 are Luminal samples. In contrast, the predictions by all the seven normalization algorithms showed poor enrichment of desired characteristics. Specifically, EB, XPN, DisTran, MRS and QD failed to predict any samples in four out of these six selected classes. GQ and DWD did predict samples in four classes; however, the enriched characteristics of the predicted samples did not agree with the original characteristics. Particularly, GQ predicted 69 samples as Class 2 but only 37 of them are ER +. It also predicted 126 Class 3 samples but only 75 of them are Luminal A samples. Over all, CL’s predictions are much more enriched with the known characteristics and it predicted more classes.

**Table 7:** Selected cancer 2000 classes and their characteristics

Cancer 2000 cluster (Selected)	Class 2	Class 3	Class 5	Class 6
Characteristics	ER +	Luminal A	ER-,Her2 enriched	Luminal Samples, ER+

**Table 8:** Comparison of cancer2000 prediction results between CL and 7 alternative cross platform normalization algorithm

Cancer2000 class	Class 2	Class 3	Class 5 ER-	Class 5 Her 2	Class 6 Luminal	Class 6 ER+
CL	36/41	24/26	20/28	21/28	24/26	26/26
EB	8/10	0/0	0/0	0/0	0/0	0/0
GQ	37/69	75/126	4/4	0/4	20/23	21/23
DWD	98/111	52/105	14/15	0/15	19/24	19/24
XPN	8/10	0/0	0/0	0/0	0/0	0/0
DisTran	0/0	256/256	0/0	0/0	0/1	0/1
MRS	0/0	29/68	0/0	0/0	0/0	0/0
QD	2/3	1/11	0/0	0/0	0/0	0/0

### Arabic breast cancer patient’s microarray data analysis

First we aimed to find genes differential expressed in Qatar breast cancer patient compare to the control sample. With two sample *t* test and adjusted *P* value set to 0.05, 116 genes showed significantly differential expression between Qatar breast cancer patients and Qatar normal breast tissue samples. We also aimed to find the genes uniquely expressed only in Aerobic species by comparing QNRF dataset with another set of breast cancer population. For comparison, dataset GSE22035 was downloaded from GEO. This dataset contains 43 Caucasian species samples. It has the same microarray platform as the QNRF dataset. Both datasets went through the same pre-process procedure and additional round of normalization was done on two datasets together. Note that this analysis was not performed on all the genes but only on the differential expressed genes detected previously. All seven cross platform normalization algorithms and quantile normalization were performed in order to detect common differential expressed gene unique to QNRF dataset. However, among all the cross-platform normalization algorithms, no common gene is reported. With Quantile normalization, 9 genes were reported but for DisTran and MRS, another set of 6 genes were reported. Although we cannot provide a consistent list of genes that differential expressed across all normalization algorithm, this 15 gene together could be our primary target of interest in future study for breast cancer in Qatar population. The PAM50 classification and Cancer 2000 classification were also reported by CL procedure (Table 9). For PAM50, the PAM50 R code classification result was also reported. PAM50 R classifies most of the QNRF samples into Lum B class, while some of them had obviously problems. For example, sample B2, B22 and B25 were both ER – and PR –, which were most likely to be Basal or Her2 subtype but PAM50 R classifies them into Lum B. On the other hand, sample B20 who is ER + and PR + was classified as Basal but is more likely to be non-Basal sample. For CL, the classified result of the above samples was much more reasonable: B2, B22 and B25 were all classified as Basal and sample B20 was classified as HER2. One interesting point is that among the 20 patients, most of the patients were identified as either Basal subtype or Her2 subtype, while only one Qatar sample was identified as Lum B. This result suggests that over all, breast cancer in Qatar population behaves more like Basal and Her2 subtypes. However, additional tests using samples from larger cohorts need to be performed to confirm this finding.

**Table 9:** Breast cancer subtype classification of QNRF

QNRF sample	ER	PR	PAM50 R call	PAM50 CL cCall	Cancer 2000 CL call
B10	+	+	LumB	Basal	cancer2000 icluster 1
B13	+	+	LumA	Basal	cancer2000 icluster 1
B14	NA	NA	LumB	Basal	cancer2000 icluster 3
B17	+	+	Normal	HER2	cancer2000 icluster 3
B18	+ +	+	Normal	HER2	cancer2000 icluster 3
B19	NA	NA	LumB	HER2	cancer2000 icluster 3
B20	+	+	Basal	HER2	cancer2000 icluster 3
B21	+	+	Lum B	Lum B	cancer2000 icluster 3
B22	-	-	LumB	Basal	cancer2000 icluster 3
B23	+	+	LumB	Basal	cancer2000 icluster 3
B24	+	+	LumB	Basal	cancer2000 icluster 3
B25	-	-	LumB	Basal	cancer2000 icluster 3
B26	+	+	Basal	Basal	cancer2000 icluster 3
B27	+	+	Basal	Basal	cancer2000 icluster 3
B2	-	-	Lum B	Basal	cancer2000 icluster 1
B3	NA	NA	Lum B	Basal	cancer2000 icluster 1
B4	+	+	Lum B	HER2	cancer2000 icluster 5
B5	+	-	Lum B	Basal	cancer2000 icluster 1
B6	-	-	Basal	Basal	cancer2000 icluster 1
B7	NA	NA	Lum A	Basal	cancer2000 icluster 1

## Discussion and Conclusions

In this paper, we proposed a novel algorithm CrossLink for cross-condition prediction of cancer classes. Unlike other normalization-based method, CL employs an unsupervised algorithm, which aims at identifying unique class-specific signatures patterns. CL was applied for cross-condition prediction of the PAM50 and Cancer2000 subtypes. In all tested datasets, CL showed robust and consistent improvement in prediction performance over other state-of-the-art normalization algorithms.

Despite its advantages, CL has limitations. First, CL is better fitted for datasets of large sample size, because CL needs to perform an unsupervised learning. It cannot be applied to individual samples separately as what a classifier would do. By the same reasoning, CL would fail when there are samples from only a single class.

Our future work includes to three directions. First, the result of the CL indicates that instead of choosing a common signature set for all subtypes classification, subtype specific signatures can lead to better robustness and accuracy for subtypes classification. Further investigation is needed to discover the biological insight of those signatures. By doing so, the subtype related function could be also discovered. Second, CL shows great potential for subtype classification in cross-condition breast cancer subtype classification. This ability could be further extended into other cancer genomic classification problems when condition specific bias presented. Third, the unique design of CL allows it bypassing the condition specific bias to achieve a robust classification accuracy. This advantage can be further extended to handle bias between different technical platforms, for example, between microarray and RNA-seq data.
